# Speciality grand challenge: next steps for coaching? Some existing and emerging issues

**DOI:** 10.3389/fspor.2024.1496079

**Published:** 2024-10-09

**Authors:** Dave Collins

**Affiliations:** ^1^ Grey Matters Performance Ltd., Stratford upon Avon, England; ^2^ Moray House School of Education and Sport, University of Edinburgh, Edinburgh, Scotland; ^3^ School of Health and Human Performance, Dublin City University, Dublin, Ireland

**Keywords:** development, profession, biopsychosocial, objectives, role

## 1 Introduction—why this paper and where am I coming from?

As a domain for study, coaching has become really popular. More undergraduate, masters' and doctoral programmes demonstrate both interest and consequential explosion of knowledge in this topic. Furthermore, coaching science receives interest from business/executive work, lifestyle and counselling. This is paralleled by increasing diversity of interest in the processes and mechanisms of coaching itself. As such, I offer this grand challenge paper as a summary of, or position statement for, where the discipline is and, more importantly, some thoughts on where it might go.

As to my own perspective? I am an experienced teacher and coach (albeit somewhat past tense), qualified across a variety of sports and have worked from grassroots to national/professional level. As such, my approach is applied and my philosophy pragmatic. In short, the best answers are often those that make a difference, although these answers should also be carefully researched! As a pracademic, I complete research and review others' work from this “does it make a difference” stance. Thus, throughout this paper you will see me raising points and asking questions which pertain to the applied implications for practise and outcome.

There is a lot to cover, so my overview will inevitably be snapshot rather than comprehensive. As a structure, I will use the biopsychosocial breakdown which, since being developed for medical applications (1) has been increasingly utilised across performance settings. To coach-ify my treatment, I will consider the bio as the motoric aspects, whilst the psych and social are presented as named.

## 2 The bio—psychomotor models of learning

Psychomotor processes are a key pillar of coaching. Knowing how to best enable skill development is core to coaching, whether this is early-stage generic movement (what some call the fundamentals) or activity specific skills and how they are deployed against strategic objectives such as team tactics. I would suggest that all these aspects are important and, therefore worthy of consideration.

Until recently, the area has been dominated by a vigorous debate between what is often described as the traditional, cognitive approach and the ecological perspective, with its associated applications such as the Constraints-Led Approach. The differences are both significant but also shifting, and I certainly can't do justice to the debate in this short paper. One (arguably over) simplified point of difference is to see the development of skill as working towards a prescribed model [for example the three-stage learning model of (2)] as opposed to a (albeit guided) discovery of a personal solution [e.g., (3)]. For many it seems that *both* approaches have something to offer [e.g., (4)], especially when considered and applied to specific contexts. Although this idea is roundly dismissed by some as a “pick and mix” approach [e.g., (5)] I don't personally see much difference to the well-developed concepts of a teaching (6) or coaching (7) styles “spectrum”. In simple terms, what methods will best achieve my set objectives in this context.

For the present purpose, however, I would highlight two important challenges that the psychomotor literature needs to address. Firstly, and especially so given the explosion of interest and usage (and sometimes unfounded claims!) around neuroscience, I would expect to see a more mechanism-driven consideration of the different positions. This may not be of direct relevance to the practising coach but this call for causation is already a feature of the mainstream neuroscience literature [e.g., Ross & Bassett (8)] and would help a great deal in solving some of the contradictions between the two established approaches. It would also help us to clarify exactly when (and when not) each approach might offer an optimum answer.

The second challenge, and one which again reflects the important role of mainstream neuroscience, is to explore and, where applicable, adopt/integrate the third way approach of active inference [e.g., (9)]. These ideas have been building in mainstream psychology for a while and, although perhaps not sufficiently well developed to form a distinct coaching approach, offer excellent insights, indeed a potential bridge, between the two “extremes” of cognitive and ecological approaches. Readers might be interested in our soon to be published text (10), aimed at both students and coaches, which offers an “it depends” based review of the (now) big *three* approaches, how they interact/contradict and how to make the best of all together.

## 3 The psycho—aims, scope and conduct of coaching

If the previous section looked at the HOW issues for modern coaches, this one considers the WHAT. The objectives and methods for coaching have never been under such critical review. Recent concerns of abuse [cf., (11)], issues over mental health, both general and sport related (12) and the dissonance that the consequent picture of objectives can create [e.g., (13)].

All these issues are important considerations for the science and practice of coaching. But have they been considered with sufficient criticality, or are we still missing some key data or perspectives? I cover some of these in the next few sections, using references from within but also out with the coaching lexicon.

### 3.1 Countering abuse—frequency and initiative-based challenge

Some form of abuse has always taken place in sport. After all it is but one facet of a society with many ills. Concerns are increasingly framed against challenge which is (coupled with support of course) essential for growth [cf (14)]. Importantly, this essential extends beyond progress in sport to general life, albeit generating some significant but transitory emotional turmoil [cf. (15)]. Perhaps we need to clarify what abuse actually is. Interestingly, overcoming challenge may be an essential component of wellbeing “well-being can be observed by the extent to which [people] are resilient, *build capacity for action, and are prepared to transcend challenges*” [my italics, Health Promotion Glossary of Terms 2021, p.10, (16)].

Our recent work applying a Rationale-Intention-Behaviour (RIB) model to coaching behaviour is one example of this. In evaluating whether the Behaviour is abusive, it's important to examine the Rationale and Intention underpinning the decision. The desires of the performer must also be taken into consideration, so long as they are sufficiently well informed and of an age to make a decision. For example, many action and adventure sports require coaches to encourage attempting skills and/or training loads which may be harmful and/or bring discomfort. In free skiing and snowboarding for example, the bond between coach and athlete is a crucial one; the athlete must trust the coach's judgement on whether they attempt a new trick or leave it for the moment (17). In other words, athlete disquiet alone is not a *sine qua non* for spotting abusive behaviour.

It is also important to recognise the pressures which these mixed agendas exert. Recent work by Voelker et al. (13) offers a good example of this, with the clashing demands of performance and aesthetics of body image against the challenges of good health for female athletes can result in neither objective being satisfied. Once again, open discussion of objectives, framed against approaches such as the RIB Model and informed by research may offer some reconciliation.

Another significant step has been the development of child and young person (CYP) initiatives: in England, this has been led by Play Their Way (18). Termed child-first coaching, the approach is presented as grounded on the UN convention on the rights of the child (UNCRC). This offers a new perspective reflecting the rights of children and young people (CYP) and is operationalized in three ways (18).

• Voice – space to share their views, which are acted on together in a meaningful way.
• Choice – they choose how they play and participate.
• Journey – they develop holistically, in their own way.

I might suggest that, whilst the UNCRC is indisputable and focused on somewhat more global issues, the genericity of play their way as a universal lead strategy is more questionable. For example, is “play their way” looking at a *sub-species* of coaching? The de-emphasis on skills and syllabi both contrast with conventional educational practice and seem at odds with CYP pursuing excellence/senior achievement [cf. (19)]. There are also questions to be raised about the primary responsibility for safety, exercised by the coach, most notably in action or adventure sports, which must surely dictate certain actions over and above the wishes of the participants.

My intention here is not to review this or any other recent initiative. What I *am* saying is that, before National Sports Organisations take a (welcome and overdue) lead in coaching, one would expect to see these evolve from clear and critically-reviewed research. Furthermore, the rationale and intention, together with the possible consequences, should have been unpacked. As such, please consider this as a call for more evidence grounded initiatives, and careful evaluation of same, as appropriate.

### 3.2 Mental health—scope and solution?

**Figure 1:**
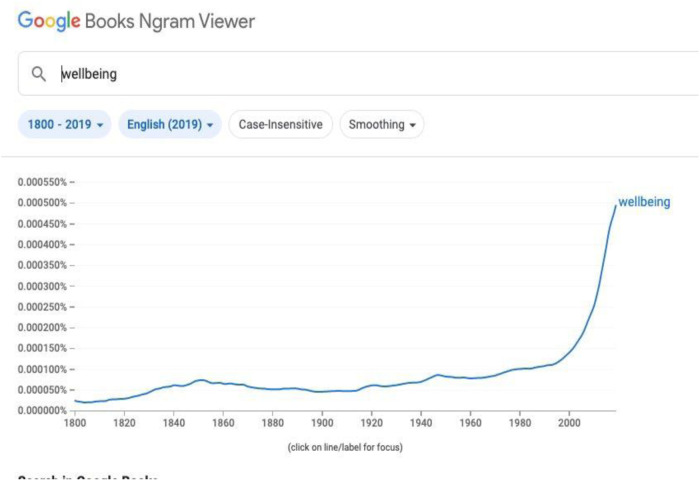
Google search data: showing the significant growth in outputs addressing wellbeing.

There is no doubt that this is a much-debated concern within our society and sport is no different. As Figure 1 shows, interest in well-being and the associated mental health issues has boomed over the last few years.

Such evidence is important: but it doesn't demonstrate that such issues are common, have grown, or are peculiar to sport. As such, data need to be carefully and critically evaluated to look at the nature as well as the numbers of self-reported and externally diagnosed/confirmed mental health issues or MHIs.

Interestingly, this issue was raised by Wessely (20), first psychiatrist president of the Royal Society of Medicine, albeit his concerns were also related to the challenges of increased awareness of MHIs on already scant resources. My point is not to challenge that people are concerned; but rather, to suggest that over awareness and medicalisation may exacerbate the situation.

Once we have accurate information on the scope, the next thing is to decide on the best combination of treatments. Again, questions need to be asked. For example, literature is raising questions about the efficacy and impact of solutions commonly in use in sporting environments. In short, is generic education useful/effective (21, 22) or perhaps even be doing more harm than good, at least in some cases [cf. (23)].

## 4 The social—consumer expectations and the “coaching milieu”

Most of the issues in the section above, the WHAT, are potentially caused or at least exacerbated, because of social expectations; in short, these may largely but perhaps unfairly drive the WHY. Once again, I will only address a couple of examples; firstly, the understandable biut perhaps ill focused emphasis on the coach as a provider of physical activity (PA).

### 4.1 Physical activity as a/the key target?

Benefits of PA are clear; so clear that many cannot understand why people don't engage. For coaches, the increasing societal importance of PA has added yet another demand; specifically, that sports have an impact on the physical health and fitness of their athletes. Fitness is an inevitable feature of engaging in sports training, so this comes down to attracting and then enthusing rather than any particular structure. These motivational factors are emerging as a feature of the coach's leadership and systemic approach, which are addressed by Social Identity Leadership [SIL – (24, 25)]. Consequently, and against the importance of PA as an agenda, work on SIL and how coaches may serve to change behaviour leading to a lifelong habit would seem desirable (26).

Additionally, it is important to contrast coach and sport led approaches with other initiatives. Often targeted at CYP, many make a mistake of thinking that activity *now* inevitably equals *activity for life*. As one example, the UK's school-based scheme, the Daily Mile (27), which sees primary kids required to run/stagger/walk a mile every day. Unsurprisingly, initial enthusiasm was high (after all *anything* is better than lessons) and several studies showed immediate benefits to fitness and cognitive measures. Notably, however, a lack of logic (how *will* this work later) and monitoring has seen this fall away. Once again, I suggest, overly simplistic and insufficiently thought through ideas to address a poorly operationalised issue. Indeed, with research suggesting that later benefits are mediated by the early development of skills and confidence [e.g., (28, 29)], the role and focus for coaches in early years becomes more than just a *provider* of PA.

### 4.2 The coach as a provider of FUN?

With clear relationships to PA and “play their way”, coaches (at least in the UK) are under increasing pressure to offer activities which engage and enthuse CYP. This has led to a focus on fun as a key measure of coaching impact. One oft cited example is the excellent and impactful research of Visek et al. (30) which has generated “fun maps”. This has, in my opinion, been seized by many as *the* blueprint which should underpin work with CYP. The importance has also overlapped into high level sport; for example, the growing meme that “a happy athlete is a fast athlete”!

In potential contrast, there is growing evidence for the positive benefits of challenge as a developmental tool, albeit offered in the right balance (14). I feel that this is another example where a lack of criticality, coupled with a failure to focus on enjoyment rather than fun *per se*, has led to mixed messages and societal pressures on coaches. The point is that, for many at all ages, improvement is the source of enjoyment, even if this s accomplished by hard, not inherently enjoyable effort [cf. (19, 31, 32)]. It might be that a more nuanced consideration of coaching (and coaching expertise) is needed, recognising that coaches, coaching *and* coaching consumers come in many shapes and sizes! After all, it may be that a fast athlete is a happy athlete!

## 5 So…what are the implications for coaching research and development?

As a summary to this Grand Challenge paper, I suggest that, despite the literature volume, several issues might be driven by evangelical zeal rather than a preferable evidence grounding. The combination of increased awareness, social media pressure and centralization of management (through institutes and NSOs for example) has raised the demand for “something” to the be done about many facets of coaching. My overarching suggestion is for a more critical approach that is built on genuine data of incidence and severity. As part of the challenge going forwards, however, I would suggest a need for research feeding into practise on the scope of the key issues which can then be genuinely addressed.

As one key underpinning, there is a need to consider the drivers coach development. Is this best accomplished by coaching leaders in specific contexts [e.g., (33)], by the social milieu within coaching [e.g., (34)] or even by the often seen as the less preferred approach of formal coach development [cf. (35)]? The answer is probably all of these and some more. However, research is needed to tease out and so promote the optimum blend.

In closing, I hope this personal view will stimulate some thought and even action in the coaching research community. I certainly look forward to the exciting possibilities which this journal can offer.
